# Rationale for Timing of Follow-Up Visits to Assess Gluten-Free Diet in Celiac Disease Patients Based on Data Mining

**DOI:** 10.3390/nu13020357

**Published:** 2021-01-25

**Authors:** Alfonso Rodríguez-Herrera, Joaquín Reyes-Andrade, Cristina Rubio-Escudero

**Affiliations:** 1 Pediatrics, Saint Luke’s Hospital, University College Dublin, Kilkenny R95 FY71, Ireland; a.rodriguezherrera@hse.ie; 2 Unidad de Gastroenterología y Nutrición, Instituto Hispalense de Pediatría, 41013 Seville, Spain; joaquinreyes@ihppediatria.com; 3 Department of Computer Languages and Systems, University of Seville, 41013 Seville, Spain

**Keywords:** celiac disease, data mining gluten free diet, gluten proteins, immunogenicity, evidence-based practice, case management, treatment adherence and compliance

## Abstract

The assessment of compliance of gluten-free diet (GFD) is a keystone in the supervision of celiac disease (CD) patients. Few data are available documenting evidence-based follow-up frequency for CD patients. In this work we aim at creating a criterion for timing of clinical follow-up for CD patients using data mining. We have applied data mining to a dataset with 188 CD patients on GFD (75% of them are children below 14 years old), evaluating the presence of gluten immunogenic peptides (GIP) in stools as an adherence to diet marker. The variables considered are gender, age, years following GFD and adherence to the GFD by fecal GIP. The results identify patients on GFD for more than two years (41.5% of the patients) as more prone to poor compliance and so needing more frequent follow-up than patients with less than 2 years on GFD. This is against the usual clinical practice of following less patients on long term GFD, as they are supposed to perform better. Our results support different timing follow-up frequency taking into consideration the number of years on GFD, age and gender. Patients on long term GFD should have a more frequent monitoring as they show a higher level of gluten exposure. A gender perspective should also be considered as non-compliance is partially linked to gender in our results: Males tend to get more gluten exposure, at least in the cultural context where our study was carried out. Children tend to perform better than teenagers or adults.

## 1. Introduction

Celiac disease (CD) is a chronic systemic immune-mediated condition that occurs in a genetically susceptible host, produced by the ingestion of nutritional gluten, the major protein component in wheat and other related cereals [1]. It is one of the most common disorders, involving around 1% of the general population and can occur at any age [2]. CD is characterized by the presence of a wide variety of CD-specific antibodies, enteropathy, gluten-dependent clinical expressions, and HLA-DQ2 or HLA-DQ8 haplotypes [3,4,5]. 

A lifetime gluten-free diet (GFD) is nowadays the only treatment for CD. Non exposure to gluten is believed to achieve mucosal recovery, resolve symptoms, and avoid the difficulties associated to non-treated CD [6]. Even though following a GFD might seem easy, it becomes a challenge in the gluten-rich Western diet. Indeed, it is increasingly recognized that many CD patients on a presumably GFD may have ongoing symptoms and/or persistent villous atrophy. Therefore, adherence to the GFD needs to be assessed to guarantee potential effects on the patient’s health condition and quality of life [5].

There is no consensus regarding the best means for assessing compliance or the optimal frequency of monitoring the GFD. Despite the availability of diverse traditional GFD adherence markers, such as dietary tests or serology, none of them are an accurate evaluation method of the dietary obedience [7,8]. As a result, finding gluten immunogenic peptides (GIP) in human urine and stools have appeared as novel markers for direct verification of GFD compliance [9,10,11]. GIP show the capacity to resist to gastrointestinal absorption and accounts for immunogenic reaction in T cells of patients with CD. Differently to traditional methods for the monitoring of GFD obedience, which only measures the consequences of GFD non-adherence, this non-intrusive method allows for a direct and quantitative evaluation of gluten exposure [11]. Using this new methodology, GIP were detected in 30–60% of CD patients on a GFD and for whom no gluten exposure was identified by dietary questionnaire or serological tests [1].

It is generally recommended that CD patients have careful long-term follow-up. Silvester et al. [12], conclude that the existing guidelines regarding CD patients follow-up proposed very different recommendations and many were not evidence-based. This study was based on gastroenterological societies and associations guidelines and recommendations by specialists obtained from MEDLINE and other Internet search engines. Javorsky, et al. [13] searched the PubMed database for works related to evidence-based guidelines on follow-up intervals for the 5 topmost chronic conditions according to the highest amount of patient attendance in 2010 in the USA (back problems, arthritis, hypertension, mental disorders, chronic obstructive pulmonary disease/asthma), with some guidelines attempting to recommend specific follow-up intervals, but not being evidence-based. They did not propose intervals based on clinical data or failed to reveal on what timing the visits were based. However, both works conclude that time frequency of visits intervals is relevant. Therefore, prospective studies appear as necessary to create cost-effective, rational, and risk-stratified guidelines for long-term follow-up of these patients [12].

Data mining can be defined as the automatic analysis of data sources to identify models representing knowledge [14]. Clinical data mining is concerned with the application of data mining techniques to clinical data [15], which in turn allows the creation of models of knowledge and aids clinical decision making [16].

In this work, we aimed at providing grounds for evidence-based follow-up frequency suggestions for CD patients, obtained by applying clinical data mining to a dataset extracted from a cohort of 188 CD patients (75% of them are children below 14 years old), whose GFD compliance was assessed. The presence of GIP in stools was used as a distinctive biomarker of GFD adherence in this series. Other variables considered were gender, age and length of ongoing GFD.

## 2. Materials and Methods

This work is based on the analysis of a retrospective dataset previously collected in a partially blinded nonrandomized, multicenter study including 188 CD patients (75% of them are children below 14 years old) following a GFD recruited between 2012 and 2014 at 13 Spanish hospitals [1]. The trial registration number is NCT02711397. This study was authorized by the ethics committee of each involved institution and informed written consent was acquired from participants over 18 years old and from parents or legal keepers for participants below 18 years old. The group under study was composed of celiac patients on GFD for at least 1 year before being included in the study. Inclusion criteria restricted enrollment to those who had an HLA-DQ2 or HLA-DQ8 haplotype test and a histologically nonstandard duodenal biopsy (grade Marsh IIIB or IIIC) at the time of diagnosis, as well as positive serum anti-endomysium IgA antibodies and/or anti-tissue transglutaminase (anti-tTG) IgA antibodies.

Adherence to GFD was evaluated by GIP detection. The concentration of GIP in feces was assessed with sandwich enzyme-linked immunosorbent assay (ELISA) [17] using the iVYDAL In Vitro Diagnostics iVYLISA GIP-S Kit (Biomedal S.L., Seville, Spain). Patients were also measured on a four-day food record dietitian review and celiac serology (tissue transglutaminase and deamidated gliadin peptide antibodies). Information regarding the date of CD diagnosis, duration of the GFD, and demographic and clinical data were also retrieved.

### Data Mining Methods

Data Mining comprises two main tasks: Prediction (supervised learning) and description (unsupervised learning) [18]. Prediction attempts to predict some or several unknown variables from other known ones. The description, however, tries to look for patterns that describe the data in a way that humans can understand.

Within the scope of prediction there are two fundamental tasks: Classification and regression. Classification tries to assign a target variable that belongs to a dataset [19] while regression aims to predict continuous values [20].

We can find a great variety of classification algorithms in the literature [19]. This work has applied the C4.5 algorithm, which according to Wu et al. [21] is one of the top 10 data mining algorithms. This algorithm is one of the best-known ones capable of building decision trees. It was implemented by Quinlan in [22] and is an extension to the ID3 [23] algorithm also implemented by him.

**Figure 1:**
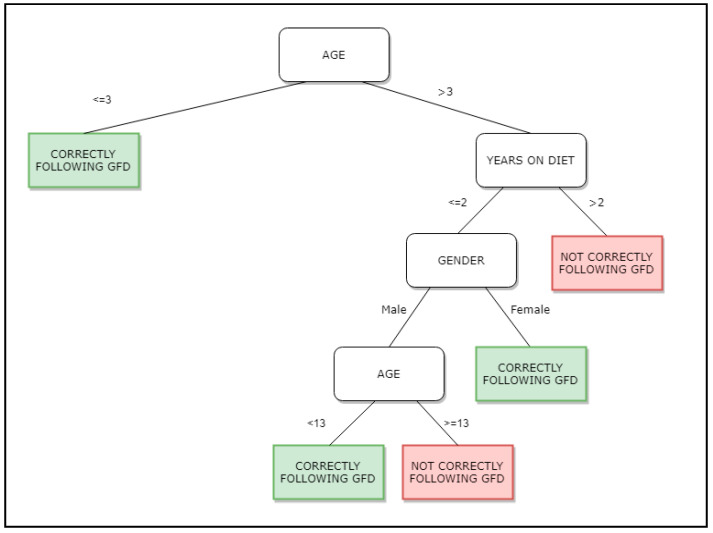
Decision tree obtained by use of the C4.5 algorithm. GFD stands for Gluten Free Diet.

Decision trees can be defined as a classification method that, given a dataset, recursively divides it into subsets using decisions specified at each branch or node in the tree. As we can see in the results shown in Figure 1, the parts of the tree are a root node (made up of all data), inner nodes (branches), and end nodes (leaves). A register from a dataset is classified by successively dividing, following the decision structure defined in the tree, and the target label is assigned to each register according to the node of the leaf on which the register is situated [24,25].

In Figure 1 we show the tree obtained with the dataset under study. Each register stores information related to the variables under study: Gender of the patient, years following GFD diet, age of the subject when collecting the sample and results positive or negative of the fecal GIP. According to the tree, if the patient is 3 years old or below, the GFD is correctly being followed, but if the age is over 3 years old and has been more than 2 years on GFD, the GFD diet is not correctly followed.

The algorithm C4.5 is described below. For a set S registers, C4.5 creates the initial tree using the divide-and-conquer strategy in this way [21,26]:

- Case 1. All the registers in S belong to the same target label or S is not big enough. Then the tree is created with only one leaf, with the target label more frequent S.
- Case 2. In other cases, select a test base on a single variable with two or more outcomes. This test becomes the root of the tree, and one branch is created for each outcome. Then, split S into subsets S1, S2… depending to the outcome for each register, and apply the same procedure recursively to each subset.

## 3. Results and Discussion

Data mining techniques are becoming very popular in clinical data analysis, as a complement to the classically used statistical analysis. Furthermore, data mining is proving to be extremely useful when the volume of data increases [27]. In this era of computer-aided health care, the management of follow-up visits and frequency with an evidence-based approach has the power to decrease costs and improve the population access to the health system [13].

The dataset collected includes four variables. The first, gender, indicates the gender of the patient, the second, years, reports the years that the patient has been on GFD, the third, age, the age of the patient when the sample was collected and finally, results, represents the result of the fecal GIP as positive or negative. This test provides information on whether fecal gluten peptides have been found, so that we can know for sure whether, or not, the subject has followed medical recommendations about not taking gluten [1].

**Figure 2:**
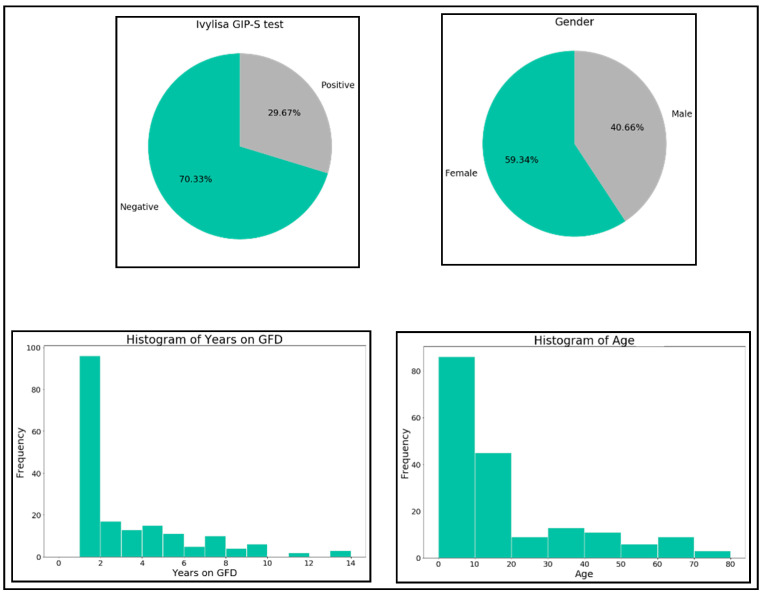
Distribution of the four variables.

Initially an exploratory analysis of the data was carried out to get an overall vision of the distribution of each of the four variables (see Figure 2).

Data showed to be unbalanced in regards to GIP (70.23% negatives and 29.67% positives) and gender (59.34% females and 40.66% males). Regarding years on GFD, most of the samples correspond to short term GFD followers. The age of the samples is in the interval (0, 20) for most of the samples. 

Data where then analyzed using the C4.5 algorithm. It was executed with different sets of parameters in order to obtain the best resulting tree in terms of area under the curve (AUC). AUC ranks in the (0, 1) interval, with 1 being the best value. It tells us how capable a model is of distinguishing a target variable, positive or negative result for the fecal GIP in our case. The resulting tree can be seen in Figure 1.

This tree is the one best representing the dataset, with an AUC value of 0.7. According to this tree, patients are more adherent following GFD as usually children below 3 years age, women with less than 2 years on GFD, and men up to 13 years old with less than 2 years on GFD. Patients not correctly following the GFD can be characterized as CD patients over 3 years old, with more than 2 years of GFD, and men with less than 2 years of GFD but more than 13 years old. This decision tree identifies patients on the GFD for a longer time, as more prone to poor compliance and perhaps needing more frequent follow-up. These results concur with the results obtained previously by Comino et al. in [1], in which they identified 13 years old as an age point for increasing dietary transgressions, as well as gender as a determining factor for these transgressions (male at certain ages are more prone to not correctly follow GFD). 

This is against the most usual clinical practice of following less the cohort of patients on long term GFD, as they are supposed to perform better.

### Current Recommendations for Frequency of Follow-Up in CD

The current clinical practice guidelines on CD do not offer a detailed background with regard to recommendations about how often patients are met for follow-up. These recommendations are based simply on suggestions of periodic visits, usually, or on an annual basis [28,29,30,31,32,33]. Despite the efforts already made to prevent or diagnose the CD early [34], there is no mention of clinical practice guidelines performing a more thorough control in adolescent patients, despite teenage being a known factor of increased risk to be exposed to gluten intake. The rationale for follow-up frequency in chronic diseases is crucial to maximize the quality of patient care. CD is a chronic disease increasing in frequency in different geographic areas [2]. In CD, non-exposure to gluten is the only “medication”. Norris et al. [35] highlighted that compliance is related to how individuals think about their personal need for a treatment in relation to their fears about the potential adverse effects. Reminders or repeated interactions with health personnel may improve compliance by building a therapeutic relationship. Hall et al. describe such process on lengthy therapies such as the one used on physical rehabilitation [36].

Some studies considering the differences linked to gender in CD have been published. Lee et al., in a study carried out by Columbia University, [37] describe gender differences as being highly significant in quality of life perceived. As examples, eating out is a problem for 20% of men and 65% of women, traveling for 18% of men and 64% of women, family life for 18% of men and 49% of women, and diet obedience, regarding the professional career, is a problem for 15% of men and 26% of women. It may be linked to a different level of awareness about the impact of gluten exposure. Despite these significant gender-specific differences, there is no differentiation on the follow up pathways [37]. Does this difference have an actual impact on long term outcomes?

None of the published guidelines consider this gender perspective. It seems sensible to assume that a better avoidance of gluten exposure will render better health outcomes. From our understanding, this is the first time a research work applies data mining to determine follow-up frequency for celiac disease. Although there have been many studies on advances in diagnosis and treatment, the volume of research on patient follow-up is significantly smaller. Scrutiny of performance of medical care can be improved by use of better data analysis. The classic methods of follow-up, serology and dietary surveys, do not present the accuracy needed to measure long term compliance. But in spite of these, most centers keep on relying on it for their decision-making process during follow up without tailoring their care to the actual profile of risk of gluten exposure. The frequency of follow-up has not been analyzed in depth and has been based on general recommendations, without individualization. Appropriate follow-up frequency must be established based on healthcare outcomes. The idea that “one size fits all” proves to be incorrect for follow-up strategies.

## 4. Conclusions

GFD treatment is very difficult to satisfy, in spite of all efforts for adherence to it, since gluten is present in most of the food we intake. The general population does not need to adhere to GFD, making the coexistence with celiac population a risk. In this work, we have been able to characterize the patients who are more adherent and those who do not correctly follow the GFD based on the four variables studied (gender, age, years on GFD, and fecal GIP).

The cohort of patients on long term GFD should have a more frequent monitoring as they tend to show higher levels of gluten exposure upon longer time on GFD. Males tend to get more gluten exposure when compared with females, at least in the cultural context where our study was carried out.

Data mining techniques applied to records could improve the identification of celiac patients who regularly transgress (voluntarily or involuntarily) whilst following a GFD. It would help to avoid more serious consequences due to persistent exposure to gluten. Timing of follow-up frequency should be different for patients newly diagnosed than for patients on the GFD for a longer period. A gender perspective should be considered as the risk off non-compliance is partially linked to gender in our results. CD management can greatly benefit from evidence-based timing of follow-up visits.

## Data Availability

Data is available upon request.
